# Mapping alternative splicing events in colorectal cancer

**DOI:** 10.1007/s12672-024-01149-z

**Published:** 2024-07-15

**Authors:** Yifeng Zheng, Guoqiang Zhong, Qiuyu Song, Haonan Zhang, Shanping Wang, Chuangzhen Lin, Chengcheng He, Mingsong Li

**Affiliations:** Department of Gastroenterology; Guangdong Provincial Key Laboratory of Major Obstetric Diseases; Guangdong Provincial Clinical Research Center for Obstetrics and Gynecology, The Third Affiliated Hospital of Guangzhou Medical University, Guangzhou, China

**Keywords:** Colorectal cancer, Alternative splicing, *CDK4*

## Abstract

Although aberrant splicing events of genes are closely related to the development and progression of colorectal cancer (CRC), the mapping of abnormal splicing events, especially alternative splicing (AS) event types and the underlying effects, remain investigational. In the present study, we analyzed a public RNA-seq database (GSE138202) and identified 14,314 significant AS events in CRC patients compared to healthy individuals. Most of the key genes such as oncogenes involved in the development of CRC have different AS event types. Moreover, the results demonstrate that certain AS events may play a significant role in the functioning of key genes involved in splicing factors and microRNAs. Furthermore, we observed that the oncogene CDK4 in CRC tends to undergo exon 2 skipping AS events, resulting in a stronger tendency for protein expression to form complexes with CCND1, thereby inhibiting the cell cycle and weakening cell proliferation, while enhancing cell migration capability. These findings not only provide new insights into the mechanism of AS in regulating CRC, but also offers a theoretical basis for targeted splicing therapy in CRC.

**Supplementary Information:**

The online version contains supplementary material available at 10.1007/s12672-024-01149-z.

## Introduction

RNA splicing, which plays a critical role in the expression of almost all human genes, involves the process of generating mature mRNA and serves as a significant contributor to protein diversity [1]. After the initial transcription of gene precursors into pre-mRNA, different mRNA splice variants are generated by selecting different splice sites. This process, known as alternative splicing (AS), leads to the production of multiple functional proteins [2]. Aberrant expressions of spliceosome elements, gene splice mutations, and abnormally RNA-binding proteins are the primary factors contributing to dysregulation of AS [3]. The core spliceosome consists of five small nuclear ribonucleoprotein (snRNP) complexes, namely U1, U2, U4, U5, and U6. These complexes are composed of functionally rich uridine-rich small nuclear RNAs (snRNAs) and over 50 different intrinsic protein components. During the process of RNA splicing, these snRNPs sequentially bind to the pre-mRNA in different complex forms, ultimately leading to the formation of a lariat structure that brings the upstream and downstream exons together, thereby facilitating the removal of introns [4]. Due to its significance in gene regulation, AS is closely associated with numerous human cancers [5].

Colorectal cancer (CRC), which ranks as the third most common cancer globally, is actively seeking improved diagnostic and therapeutic approaches [6]. Recent reviews have indicated that abnormal AS events in numerous genes significantly affect multiple facets of CRC, such as onset, proliferation, invasiveness, apoptosis resistance, immune response, angiogenesis, metabolic reprogramming, and drug resistance [3, 7]. Abnormal AS events primarily produce aberrant splicing variant transcripts through splicing, altering protein translation sequences and resulting in proteins with different structures and functions, thereby impacting CRC. For example, the tumor suppressor gene APC effectively inhibits the growth of colon tumor cells, but its splicing variant 0.3 APC lacks tumor-suppressing activity due to changes in the protein's conserved domains, weakening its interactions with other proteins and promoting tumor progression [8]. When MKNK2 is spliced into MKNK2b instead of MKNK2a, it enhances CRC tumor proliferation [9]. Additionally, the generation of splicing variants that affect drug resistance consequently impacts CRC drug resistance. For instance, Osteopontin (OPN) splicing isoform OPNc can stimulate cell survival under drug-induced microenvironmental stress post 5-FU treatment in colon cancer cells [10]. Splicing variants of SYK, SYK(L) and SYK(S), increase CRC cell sensitivity to 5-FU [11]. Splicing variant LGR 5FL-positive cells exhibit low proliferative activity and resistance to antitumor drugs [12]. Therefore, in recent years, new targeted therapies have emerged for CRC, with a specific focus on splicing variants as therapeutic targets [5]. This treatment approach offers enhanced tumor specificity and presents a safer and more controllable option for the treatment of CRC.

The main regulators of AS in tumors are splicing factors (SF), including the serine-arginine (SR) protein family (e.g., SRSF2), heterogeneous nuclear ribonucleoprotein (hnRNP) family (e.g., hnRNPA1), and other RNA-binding protein (RBP) family members (e.g., RBM5, RBFOX2) [13]. Alterations in these SF may lead to various abnormal AS events, resulting in the production of proteins with different functions, thus exerting an impact on CRC. Carcinogenic splice variants of common cancer genes such as *KRAS* [14, 15], *BRAF* [16], and *CDK4/6* [17] are found to be positively regulated in the development and progression of CRC, while tumor suppressor genes like *APC* [18] and *TP53* [19] are negatively regulated. Among these, cyclin-dependent kinases 4 (CDK4) and 6 (CDK6) are pivotal in mediating the transition of cells into the S phase, essential for the initiation, growth, and maintenance of numerous cancer types [20]. This process is predominantly driven by the Cyclin D-CDK4/6 complex [21], with studies indicating that abnormalities in both *CDK4* [22] and *CCND1* (Cyclin D1) [23] are linked to CRC. When the levels of D-type cyclins increase, CDK4 can form a complex with CCND1 and p27 protein, thereby facilitating the phosphorylation of retinoblastoma tumor suppressor protein (Rb) and other substrates [24]. This process partially alleviates the inhibitory impact of Rb on the activity of E2F family transcription factors, thus promoting cell cycle progression [25]. This represents an important target for the anticancer effects of clinically utilized *CDK4/6* inhibitors. Furthermore, in recent years, increasing evidence has shown that microRNAs (miRNAs) exert a pivotal role in various signaling pathways involved in the initiation and progression of CRC [26, 27]. These miRNAs are recognized as key players within numerous target pathways associated with CRC pathogenesis [28, 29].

Therefore, it is essential to focus on the aberrant AS events associated with oncogenes (especially *CDK4*), splicing factors, and microRNAs, in order to further investigate the underlying mechanisms of CRC. Additionally, a comprehensive analysis of the distribution patterns, potential roles, and associated pathways of AS events in CRC may provide novel approaches and targets for early diagnosis and targeted therapy of CRC.

## Materials and methods

### Data preparation

The RNA-seq data were obtained from the National Center for Biotechnology Information (NCBI) Gene Expression Omnibus (GEO) dataset (GSE138202). High-throughput sequencing was employed to conduct expression profiling, with sequencing performed on an ILLUMINA (HiSeq X Ten, yielding a mean run of 45.16 M spots. The mean size for the sequencing file includes 15.1G bases according to the SRX6926045 project detail. RNA-depleted total RNA sequence was performed to profile transcriptome expression using 8 normal and 8 human colorectal cancer tissues. The Fastq files were aligned to the human Hg19 genome using bwa-0.7.17, and the indexed.bam files were subsequently generated by Samtools (1.9) for further analysis. The data underwent quality control assessment using FastQC (version 0.11.9).

### Identification of significant AS events

The identification and quantification of AS events were performed using MISO version 0.5.4 on a total of 16 samples. A read length of 150 was applied, while keeping other parameters at their default settings in MISO. Computational analysis relied on the values generated by MISO’s “pe_utils” tool specifically designed for paired-end sequencing data analysis. The level of AS events was determined based on the percent spliced in (PSI). The Mann–Whitney U test was conducted on the two groups of samples based on the PSI values, resulting in a p-value. AS events with a p < 0.01 were deemed statistically significant, indicating significant differences between the two sample groups. The visualization of AS events (sashimi plot) was generated using the “sashimi_plot” tool within MISO.

### GO (gene ontology) and KEGG (kyoto encyclopedia of genes and genomes) pathway analysis

The GO and KEGG pathway analysis was performed using the OmicShare tools, which is a freely accessible online platform for data analysis (https://www.omicshare.com/tools).

### Analysis of differentially expressed genes (DEGs) and Gene set enrichment analysis

The TCGA RNA-seq datasets were downloaded for tumors comprising COAD and READ. Gene Expression Quantification profiles (STAR-Counts) were assessed to compare the high and low *CDK4* expression groups using the R package “DESeq2 (Version 1.38.3)” in order to validate differentially expressed genes (DEGs). Significance was determined based on differences with a | log2 fold change|> 0.5 and an adjusted P-value < 0.05.

The “ClusterProfiler (version 4.6.2)” package was used to perform GO enrichment and KEGG pathway analysis of the *CDK4*-related DEGs. Subsequently, the result of functional enrichment analysis was visualized using the “ggplot2 (version 3.4.4)” package.

### Cell culture

Human CRC cell lines (RKO, HCT116, SW480, CACO2, HT29) and normal colonic epithelial cells (NCM460) were cultured for the experiments. Cells were maintained in Dulbecco’s Modified Eagle Medium (DMEM) (Gibco) supplemented with 10% certified fetal bovine serum (FBS) (ExCell Bio), in a humidified incubator at 37 °C with 5% CO2.

### Small interfering RNA transfection

Transfection of siRNA was performed using polyplus jetPRIME^®^ reagent. Control and CDK4 exon2 (CDK4E2) siRNA (GenePharma, Shanghai, China) were transfected into RKO cells according to the manufacturer's instructions. The CDK4E2 siRNA sequence used in the study were “Forward CUUGAUCUGAGAAUGGCUATT” and “Reverse UAGCCAUUCUCAGAUCAAGTT”.

### Total RNA isolation and quantitative real-time PCR (qRT-PCR)

Total RNA was extracted using the MolPure^®^ Cell RNA Kit according to the manufacturer’s instructions. Reverse transcription of RNA into cDNA was performed using the Hifair^®^ III 1st Strand cDNA Synthesis SuperMix. Real-time fluorescent quantitative PCR analysis was then conducted using the Hieff UNICON^®^ Universal Blue qPCR SYBR Master Mix. Glyceraldehyde-3-phosphate dehydrogenase (GAPDH) was used as the loading control. Data analysis was performed using the 2^-∆∆Ct^ method. The primer sequences used were (5′–3′): CDK4SE2-F: GTGGAAACTCTGAAGCCGAC, CDK4SE2-R: ACATCTCGAGGCCAGTCATC. The primers were purchased from the GenePharma.

### CCK-8 cell proliferation assay

Cells were plated at a density of 1000 cells per well in 96-well plates. After 24 h, CCK8 reagents (GLPBIO) were added at a concentration of 10% (90 µl medium with 10 µl CCK8). Following a 2-h incubation, the absorbance of each well was measured at 450 nm using a microplate reader. This experiment was carried out over a period of six consecutive days, with three replicates for each experiment.

### Migration assays

Transwell chambers with 8 μm pore size membranes (Corning Incorporated, Corning, NY, USA) were employed for conducting cell migration and invasion assays. Cells suspended in serum-free culture medium (1 × 10^5^ cells/200 μl) were added to the upper chamber of the Transwell, while 500 μl of culture medium containing 10% FBS was added to the lower chamber. The chambers were then incubated at 37 ℃ for 36–48 h. After incubation, the cells were harvested, fixed in 4% paraformaldehyde for 20 min at room temperature, and stained with hematoxylin. The membranes were mounted onto glass slides, and cell counting was performed in 5 randomly selected fields using a microscope. For wound healing assays, cells with 70–80% confluence were seeded in a 6-well plate and a scratch was made using the tip of a pipette at time 0. The cells were subsequently cultured in DMEM, and photographs were taken at 24- and 48-h post-scratch. The gap distance was quantified using Image J software. Each experiment was repeated three times.

### Flow cytometry

After washing the cells once with PBS and centrifuging at 2000 rpm for 5 min, the cells were collected, and the cell concentration was adjusted to 1 × 10^6/ml. Subsequently, 1 ml of the cell suspension was taken, and the supernatant was removed after centrifugation. The cells were then fixed with 70% ethanol at 4 ℃ overnight. After washing away the ethanol with PBS, PI staining was performed according to the manufacturer’s protocol. The samples were analyzed using a flow cytometer. Data analysis was performed using ModFit LT 5.0.

### Statistical analyses

The variances among different groups were compared using similar statistical methods. All data represent at least three independent replicates and are presented as mean ± SE. Statistical analysis was performed using SPSS 26 software, with two-tailed tests applied. The Mann–Whitney U test was employed for analyzing bioinformatics raw data, while the t-test was used for normally distributed data. Statistical significance was denoted as follows: *p < 0.05, **p < 0.01, ***p < 0.001.

## Results

### AS events in CRC

**Fig. 1:**
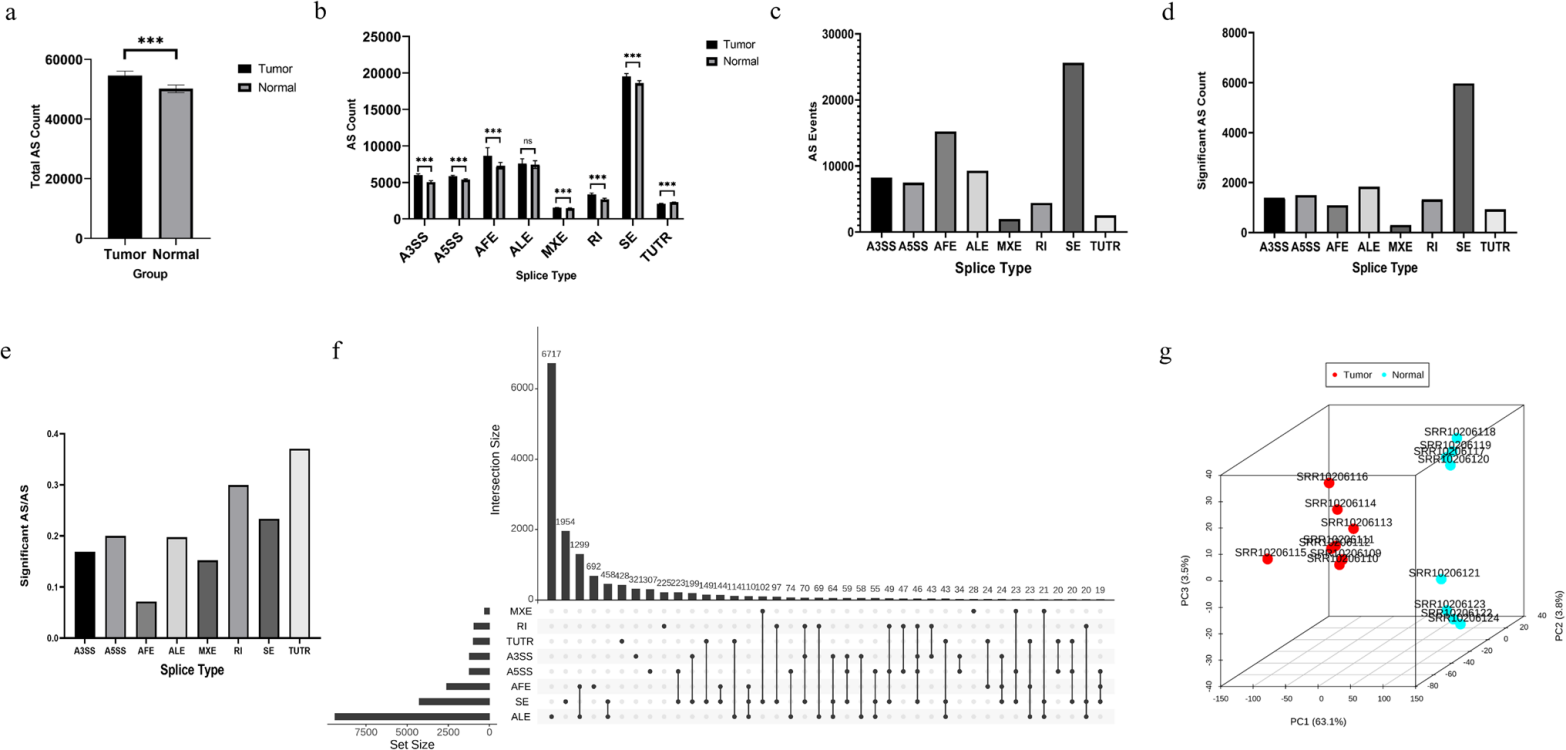
Distribution and differential expression of AS events identified in CRC and normal tissues. **a** Total number of identifiable AS events in tumor group and normal group. **b** The number of AS events for eight AS types in the tumor group and the normal group. **c** Significant AS event counts in different splice types. **d** The distribution of significant AS events among the eight splice types. **e** The significance rate of AS events in each AS type. **f** The distribution and intersection of genes involved in significant AS events among the eight AS types. **g** Principal components analysis (PCA) for the AS events in 16 samples

In the analysis of GSE138202 data for the identification of AS events, we have identified eight distinct types: alternative 3′ splice site (A3SS), alternative 5′ splice site (A5SS), alternative first exon (AFE), alternative last exon (ALE), mutually exclusive exons (MXE), retained introns (RI), skipped exon (SE), and tandem 3′ untranslated region (TUTR). Subsequently, we extracted the count of detectable AS events and their corresponding percent-spliced-in (PSI) values for each sample. In terms of the number of detectable AS events alone, a significant difference was observed between the tumor group and normal groups (P < 0.01), with the former exhibiting a higher number of AS events (Fig. 1a). Furthermore, upon comparing the number of detected AS events across different types of alternative splicing, it was observed that, except for ALE events, there were significant differences between the tumor group and the normal group for all other types of alternative splicing (P < 0.01). Additionally, among these alternative splicing types that showed significant differences, except for TUTR, which exhibited a lower number of AS events in the tumor group, the remaining types demonstrated a higher number of AS events in the tumor group (Fig. 1b). In this set of 16 samples, a total of 74,654 distinct AS events were identified. Among them, SE events were found to be the most abundant with 25,592 occurrences followed by AFE events with 15,217 occurrences. The least frequent events observed were MXE and TUTR with only 1948 and 2492 events, respectively (Fig. 1c).

Subsequently, based on the PSI values, we conducted a Mann–Whitney U test to identify significant AS events in CRC, resulting in a total of 14,314 occurrences being identified (Supplementary table S1). Among these events, SE events remained the most abundant with 5971 occurrences, followed by ALE with 1832 occurrences. The least frequent major event was MXE, which only had 297 occurrences (Fig. 1d). However, in terms of significance ratios, despite having lower event counts compared to others, TUTR and RI events exhibited the highest significance ratios at 37.08% and 29.97%, respectively. This indicates that approximately one-third of their respective AS events were deemed significant. Conversely, AFE events had the lowest significance ratio at only 7.15% (Fig. 1e).

Next, we conducted a gene count analysis for these significant AS events and identified a total of 15,023 gene symbols were identified. The distribution and overlap of these gene symbols across the eight splicing types revealed that ALE events exclusively involved the highest number of gene symbols, with 6717 genes. This was followed by SE events exclusively involving 1954 genes. Among all two or more type intersections, the highest number of genes simultaneously involved in both ALE and AFE events (1299 genes), as well as in both ALE and SE events (458 genes). In contrast, splicing types with fewer significant AS events such as MXE TUTR, and RI had a lower number of associated gene symbols (Fig. 1f).

### Principal component analysis (PCA)

To characterize the AS events between tumor and normal samples, we conducted PCA on the MISO results of the 14,314 statistically significant AS events. The first, second, and third principal components effectively captured the underlying biological differences between CRC patients and the control group (Fig. 1g).

### GO and KEGG pathway of AS effect on CRC

**Fig. 2:**
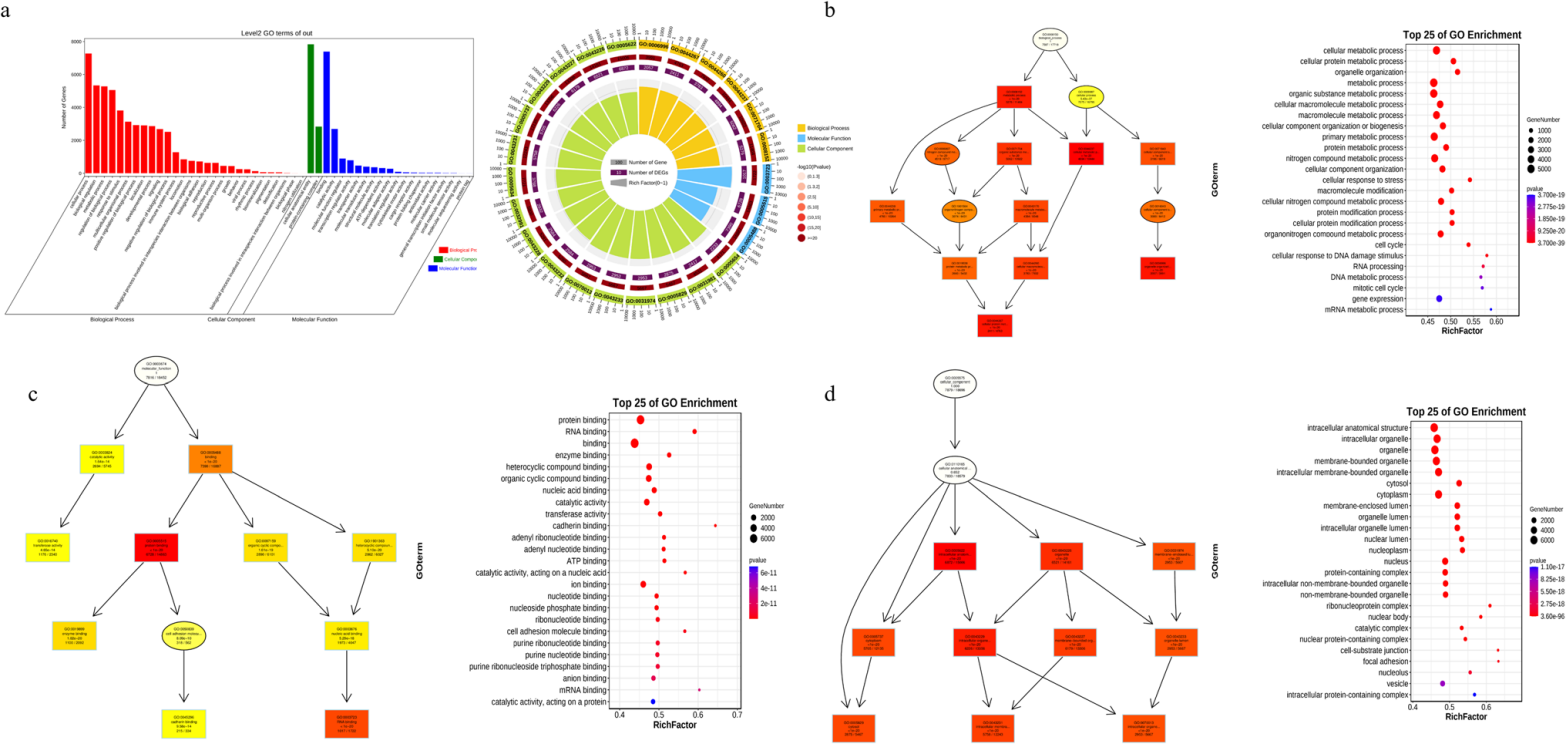
The Gene Ontology (GO) enrichment analysis results of genes involved in significant AS events between CRC and normal samples. **a** Level 2 GO terms bar chart and enrichment circle plot for significant AS-related genes. **b** Biological Process GO enrichment network diagram and top 25 enriched terms bubble plot. **c** Molecular Function GO enrichment network diagram and top 25 enriched terms bubble plot. **d** Cellular Component GO enrichment network diagram and top 25 enriched terms bubble plot

We conducted Gene Ontology enrichment analysis on the genes involved in these significant AS events (Fig. 2a), resulting in enrichment results for three ontologies: Biological Process (Fig. 2b), Molecular Function (Fig. 2c), and Cellular Component (Fig. 2d). The figures present the top 25 most significant GO terms in each ontology (P < 0.01). The enriched Biological Process terms include cellular-level metabolic processes such as “cellular protein metabolism” and “macromolecule metabolism” (Fig. 2b). The most enriched Molecular Function terms encompass “protein binding”, “RNA binding”, and “enzyme binding” (Fig. 2c). In terms of Cellular Component, highly enriched categories are “intracellular anatomical structures”, “organelles”, and “intracellular organelles” (Fig. 2d).

**Fig. 3:**
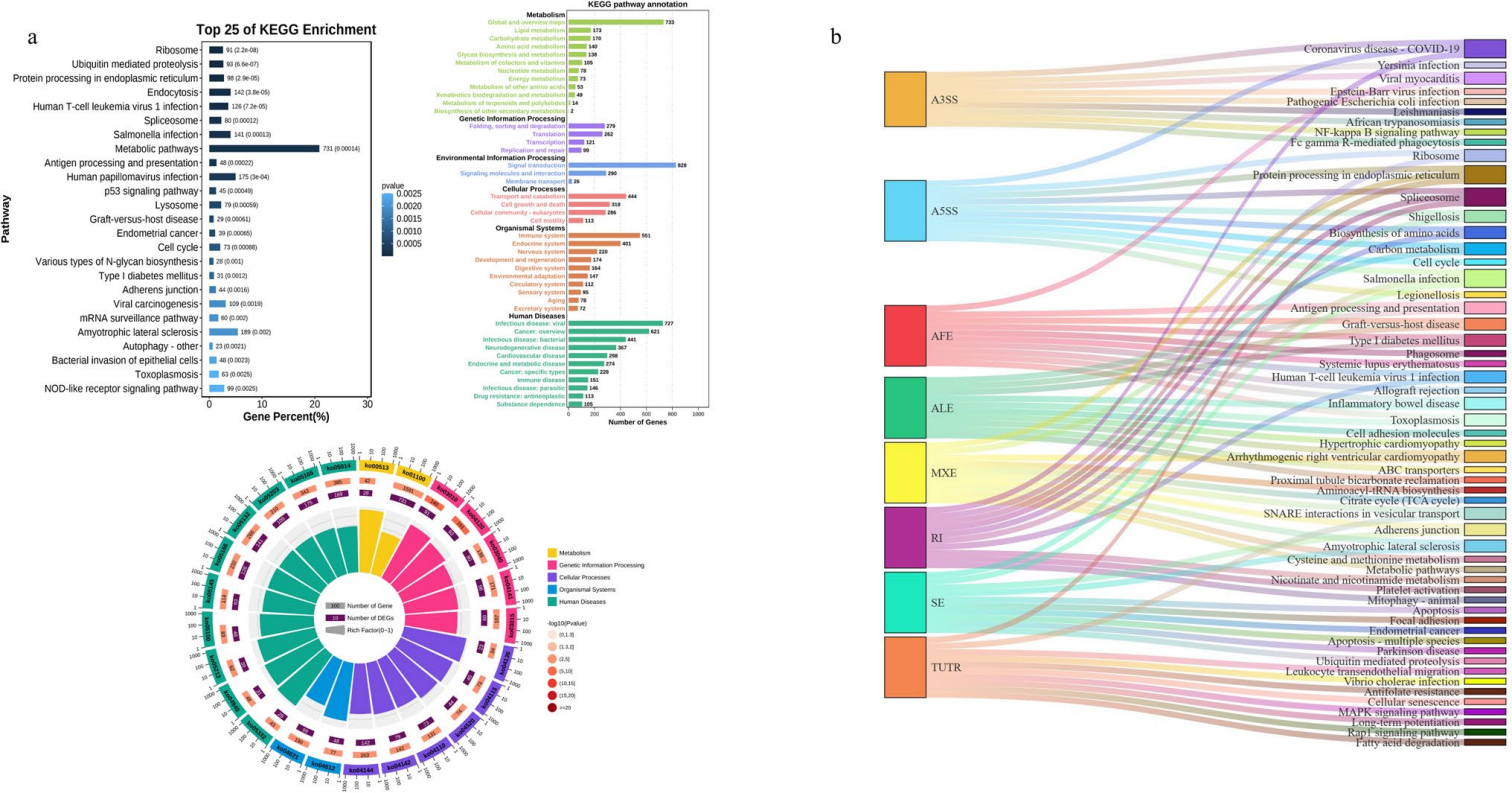
The results of Kyoto Encyclopedia of Genes and Genomes (KEGG) pathway enrichment analysis for genes involved in significant AS events, as well as the enrichment pathways and intersections among various AS types. **a** The figure shows the top 25 most enriched KEGG pathways for AS-related genes, as well as the most enriched pathways and the number of enriched genes in 6 categories of KEGG pathway annotation. The pie chart intuitively displays these results. **b** The Sankey diagram illustrates the intersections of KEGG enriched pathways among 8 types of alternative splicing

We conducted KEGG pathway enrichment analysis and obtained the top 25 most relevant pathways (P < 0.01) (Fig. 3a). The most enriched pathways include “Ribosome”, “Ubiquitin-mediated proteolysis”, “Protein processing in endoplasmic reticulum”, and “Endocytosis”. Additionally, we observed enrichment of pathways related to splicing or cancer such as “Spliceosome”, “p53 signaling pathway”, and “Viral carcinogenesis”. Then, we categorized these pathways into six groups: metabolism, genetic information processing, environmental information processing, cellular processes, organismal systems, and human diseases. Among these six KEGG pathway annotations, “Global and overview maps”, “Signal transduction”, “Infectious disease: viral”, “Cancer: overview”, “Immune system”, and “Transport and catabolism” have the highest number of enriched genes.

The genes associated with each splicing type were subjected to individual KEGG pathway enrichment analysis, resulting in the identification of the top 10 most relevant pathways for each type (Fig. 3b). We observed that certain pathways, such as “Covid-19”, “Spliceosome”, “Salmonella infection”, and “Inflammatory bowel disease”, exhibited simultaneous enrichment across multiple splicing types.

### CRC-related genes, splice factors and microRNAs involved in AS events

**Fig. 4:**
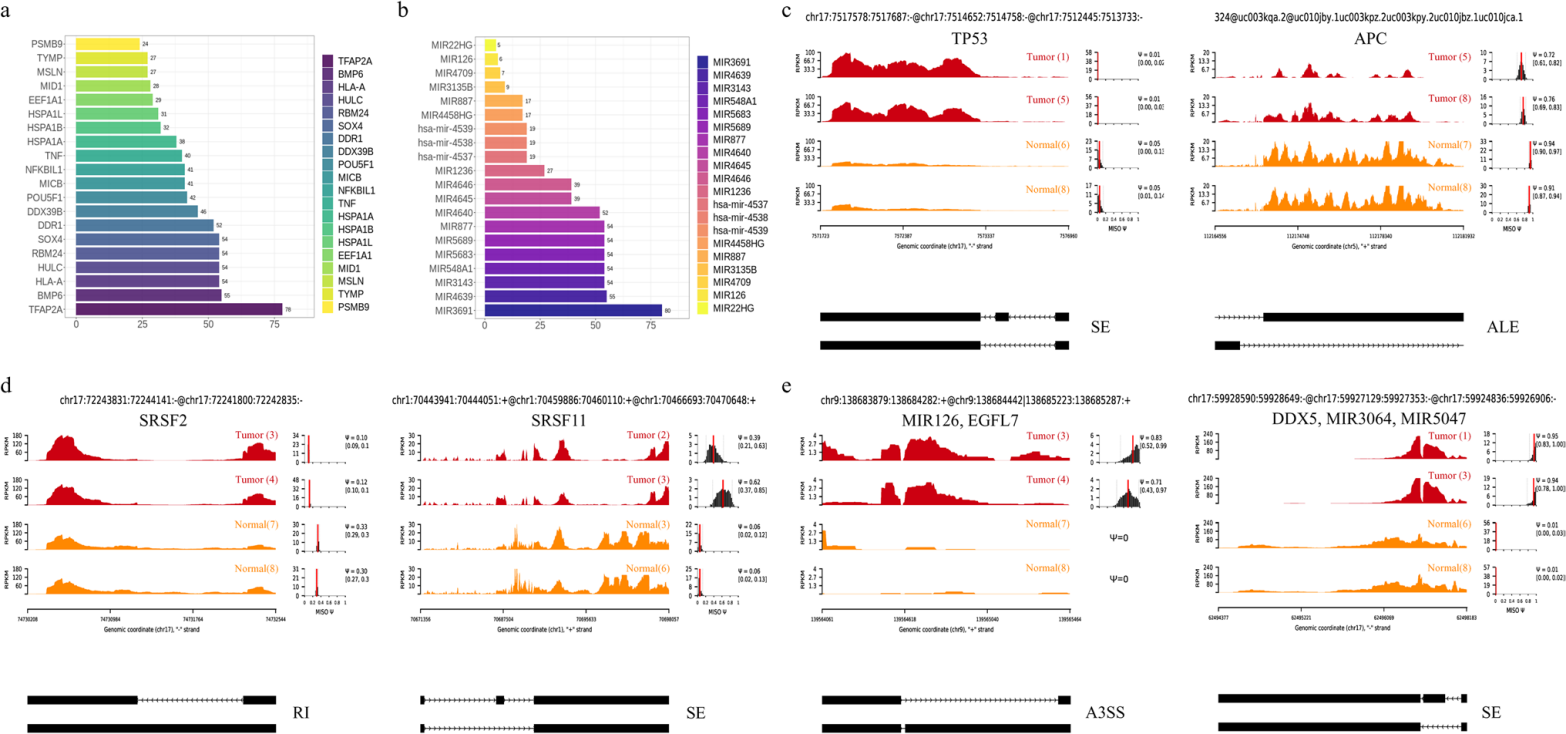
Visualization of AS analysis in 16 samples. **a** Top 20 CRC-related genes with the highest involvement in significant AS events. **b** Top 20 microRNAs with the highest involvement in significant AS events. **c** Visualization of significant AS events on the common tumor suppressor genes TP53 and APC in CRC. **d** Visualization of significant AS events on the splice factors SRSF2 and SRSF11, which are associated with CRC. **e** Visualization of significant AS events involving CRC-related microRNAs

The genes and microRNAs associated with CRC, which have been previously reported, are of particular interest to us due to their involvement in AS events in CRC samples [26]. Therefore, we have compiled the top 20 CRC-related genes (Fig. 4a) and MicroRNAs (Fig. 4b) with the highest number of AS events involved. Additionally, we have selected the *APC* gene and *TP53* gene, closely associated with CRC, and visualized their respective significant AS events (Fig. 4c). By comparing CRC samples to normal samples, distinct AS patterns were observed in these classical CRC-related genes. The occurrence of aberrant AS is regulated by splicing factors present on the spliceosome, with proteins encoded by the Serine/Arginine-Rich Splicing Factor (*SRSF*) gene family being integral components of the spliceosome machinery. Therefore, we specifically focused on *SRSF2* and *SRSF11* genes, which have been previously shown to be associated with CRC and visualized their significant AS events as well (Fig. 4d). Additionally, we also paid attention to the A3SS event involving both *MIR126* (known to be associated with CRC [30]) and *EGFL7*, as well as the SE event involving *DDX5* (a known splicing regulator [31]), *MIR3064*, and *MIR5047* (Fig. 4e).

### *CDK4* skip exon 2 AS events are more prone to occur in CRC

**Fig. 5:**
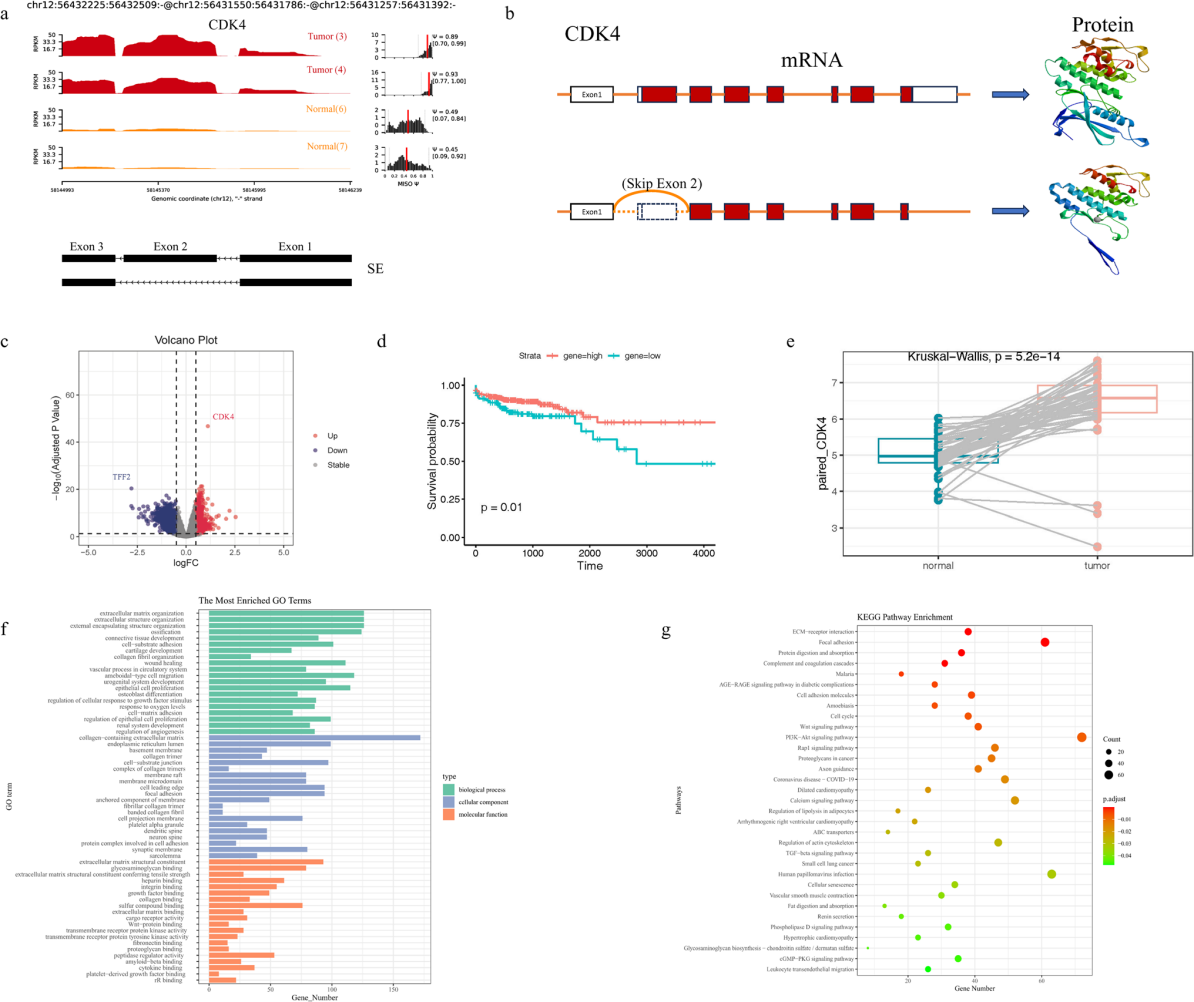
CDK4 participated in the development of CRC. **a** Visualization of the AS event involving exon 2 skipping in CDK4. **b** Protein structures of the CDK4-001 and the CDK4-007 transcripts. **c** Volcano plot of the DEGs between patients with high and low levels of CDK4 expression. **d** CDK4 expression levels in COAD and READ and matched normal tissues in TCGA. **e.** Kaplan–Meier survival curves of COAD and READ patients with high and low CDK4-expressing tumors. **f**, **g** GO and KEGG enrichment analysis results of CDK4-related DEGs

As an oncogene in CRC, *CDK4* plays a crucial role in the initiation, progression, and cell cycle regulation of CRC. Our data analysis results reveal a significant difference in the PSI values of *CDK4* skip exon 2 AS event between the CRC group and the normal group (Fig. 5a). Moreover, higher PSI values are observed in the CRC group, indicating an increased propensity for this AS event to occur in CRC.

Based on the data obtained from the Ensembl Genome Browser (https://grch37.ensembl.org/index.html), it was determined that the *CDK4* skip exon 2 AS event generates the CDK4-007 transcript (ENST00000546489.1) lacking exon 2, while the CDK4-001 transcript (ENST00000257904.6) retains the canonical transcript of exon 2. Subsequently, we employed Swiss-Model (https://swissmodel.expasy.org/) to predict the protein structures of these two transcripts (Fig. 5b). Interestingly, based on protein prediction results, we found that the proteins generated by these two transcripts have different N-terminal structures. The predicted protein structure for CDK4-007 transcript is derived from the p27/CDK4/CCND1 complex [24], while the predicted protein structure for CDK4-001 transcript is derived from the CDK4/CCND3 complex [32]. This suggests that there may exist notable differences in the abundance or ratio of CDK/CCND complexes between CRC and normal tissues.

Furthermore, we utilized DSEeq2 in R to analyze the TCGA RNA-seq data and identified the DEGs between the high and low CDK4 expression groups, using a criterion of |log FC|> 0.5, and adjusted P-value < 0.05 (Fig. 5c). The subsequent GO and KEGG enrichment analyses revealed that *CDK4*-related genes exhibited significant enrichment in various biological processes including cell adhesion, migration, proliferation, as well as signaling pathways such as Wnt signaling pathway, PI3K − Akt signaling pathway, Rap1 signaling pathway, etc. in COAD and READ (Fig. 5f and g). We also noticed that although CDK4 is expressed at higher levels in CRC patients, those who express high levels of CDK4 show a higher probability of survival compared to those with low expression levels (Fig. 5d and e).

**Fig. 6:**
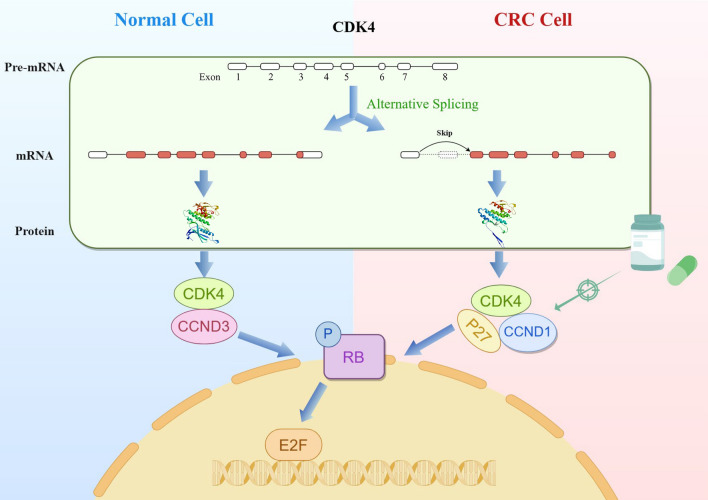
CDK4 is more prone to undergo skipping of exon 2 in CRC, resulting in the production of protein isoforms that differ from those in normal tissues. These isoforms form distinct CCND/CDK4 complexes, which in turn promote RB phosphorylation, leading to the release of E2F transcription factors and facilitating cell cycle progression

Therefore, it can be inferred that the higher occurrence of the CDK4-SE2 alternative splicing event in CRC leads to an increased production of the CDK4-007 transcript and subsequent formation of the p27/CDK4/CCND1 complex. In contrast, normal individuals exhibit a higher abundance of the CDK4-001 transcript and formation of the CDK4/CCND3 complex. Consequently, these differential complexes involving CDK/CCND may result in varying levels of downstream RB phosphorylation and release of E2F transcription factors, ultimately exerting different degrees of influence on the cell cycle (Fig. 6).

### Exon 2 skipping AS events in CRC weaken cell proliferation but enhance cell migration capability

**Fig. 7:**
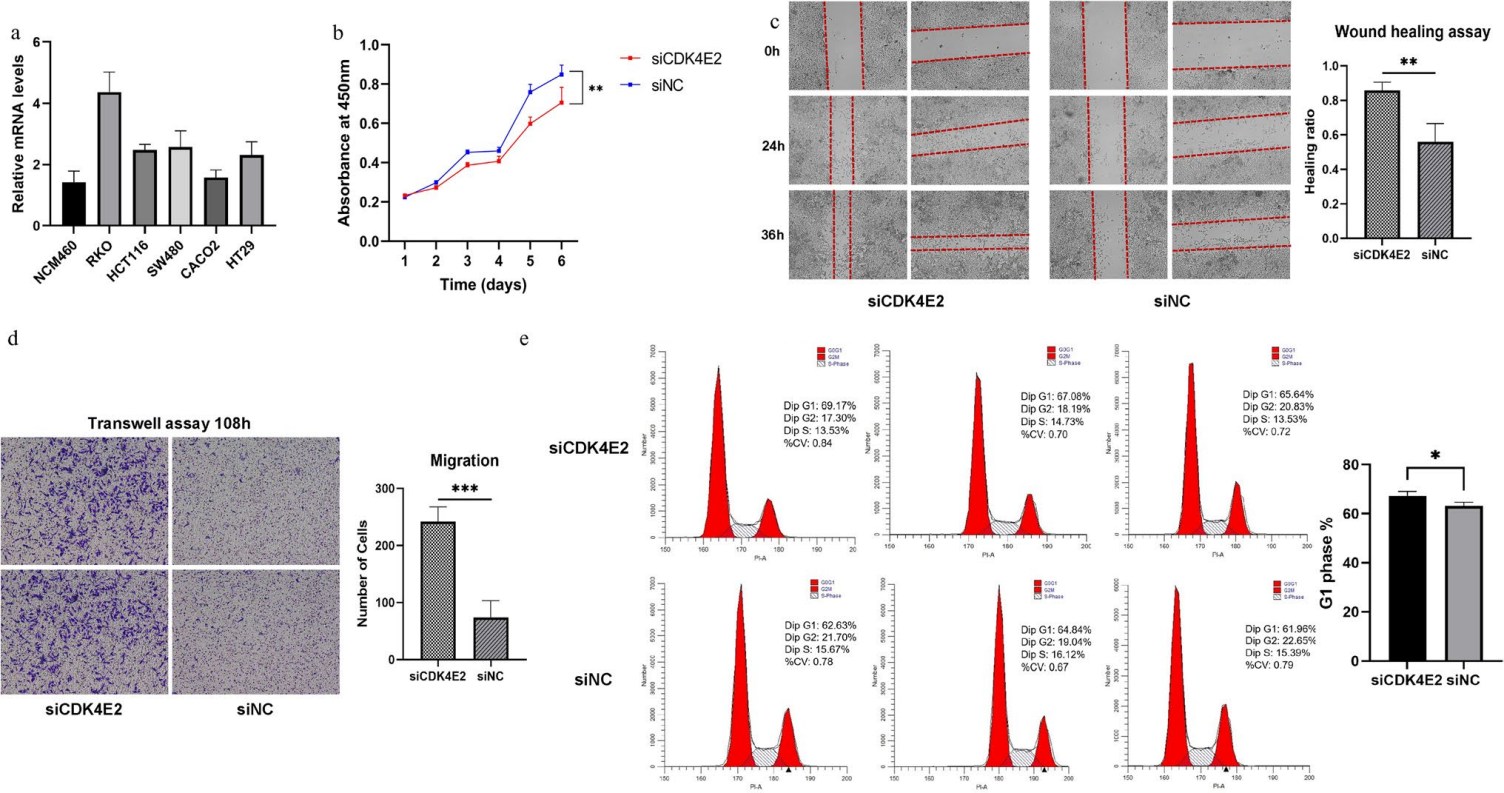
After knocking down exon 2 of CDK4 using siRNA, tumor cells exhibited weakened proliferation ability and enhanced migration capability. **a** The bar chart was used to show the relative mRNA expression levels of CDK4SE2 in RKO, HCT116, SW480, CACO2, HT29 and NCM460 by the real-time PCR. **b** The impact of knocking out exon 2 of CDK4 on the proliferation ability of RKO cells was investigated using the CCK-8 assay. **c** The Wound healing assay was conducted to investigate the effect of siCDK4E2 on the migration ability of RKO cells. **d** Transwell migration assay was performed to observe the migration ability of RKO cells after siRNA interference with CDK4E2. **e** Flow cytometry was used to analyze the cell cycle of RKO/siNC and RKO/siCDK4E2 cells. PI staining was used in each group. Bar graphs were utilized to display the G1 phase of the cell cycle. Each experiment was repeated three times

We detected the expression of CDK4SE2 transcripts in five CRC cell lines (RKO, HCT116, SW480, CACO2, HT29) and the human normal colon cell line NCM460. The results showed that, except for CACO2, the expression of this transcript was higher in the other CRC cell lines compared to normal cells, with the highest expression observed in RKO cells (Fig. 7a). Therefore, we chose to proceed with experiments using the RKO cell line. Firstly, we transfected RKO cells with siRNA specifically designed against exon 2 of CDK4, followed by conducting tumor cell function-related experiments (Fig. 7b–e). The results of the CCK-8 cell proliferation assay showed that, following the knockdown of exon 2 in CDK4 using siRNA, the proliferation capacity of CRC cells was significantly reduced compared to the siNC group (Fig. 7b). We then further investigated the impact of knocking down CDK4E2 on the migration ability of CRC cells. Both the wound healing assay and Transwell migration assay demonstrated a significant enhancement in cell migration capability following the disruption of CDK4E2 in tumor cells (Fig. 7c and d). The results of the flow cytometry cell cycle experiment also confirmed that, following siRNA interference with CDK4E2, more cells were arrested in the G1 phase, indicating cell cycle inhibition, consistent with the results of the CCK-8 assay (Fig. 7e). Therefore, our experimental results indicate that the exon 2 skipping AS event of CDK4 in CRC, while weakening tumor cell proliferation, significantly enhances cell migration.

## Discussions

In recent years, with the increasing research on post-transcriptional regulation of CRC, numerous studies have demonstrated that AS is a critical feature of CRC transcriptome variation and closely associated with the occurrence and development of CRC [33–37]. Therefore, we conducted a comprehensive analysis study of differential AS events between CRC and normal tissues based on public databases. Based on our experimental results, we have observed that CRC exhibits a higher frequency and complexity of AS events compared to normal individuals. Among these AS events, SE events are the most prevalent and abundant in CRC. These splicing events result in the production of many truncated transcripts, leading to diverse functions of truncated proteins when compared to normal splicing. Previous studies have indicated that many of these truncated isoforms can affect G protein-coupled receptors (GPCRs) [38] or regulate the DNA damage response (DDR) [39], thereby influencing fundamental biological processes and disease development. Meanwhile, although the number of RI and TUTR types of splicing events in CRC is relatively low compared to other types, they exhibit the highest significance rates. More than 30% of these two types of splicing events show significant differences between CRC and normal tissues. Additionally, PCA analysis demonstrated clear distinguishability between CRC and normal groups by utilizing different AS events as principal components, indicating the reliability of AS events as comprehensive features for CRC patients. These findings suggest that aberrant and dysregulated AS events may be one of the important contributing factors in the occurrence and progression of CRC.

SF, serving as a regulator of AS, is implicated in the pathogenesis of tumor-related alterations in RNA splicing. SF mainly consists of the SR protein family, hnRNP family, and other RBP families. Several SFs have been identified to possess oncogenic properties, such as SRSF1, SRSF3, SRSF6, HNRNPA2/B1, or HNRNPH. Conversely, certain SFs, including QKI, RBM5, RBM6, and RBM10, act as tumor suppressors and exhibit anti-cancer characteristics [13]. Our research has also revealed that many SF genes undergo AS events during their own transcription, resulting in the generation of different isoforms. Aberrant AS and/or mutations in these SFs can lead to erroneous gene splicing, thereby giving rise to pathogenic gene and protein isoforms, ultimately impacting tumor development [40]. Therefore, SFs represent a novel target for CRC therapy. For instance, when we identify a splice variant that contributes to CRC progression but is difficult to directly target, we can indirectly reduce the production of this oncogenic isoform by targeting the SFs involved in regulating its AS. This approach can effectively control the progression of CRC.

Moreover, the selection of AS sites is controlled by the dynamic formation of protein complexes on pre-mRNA, with the phosphorylation of SFs and RBPs regulating their assembly on precursor mRNA, thereby modulating the AS process. Recent studies have increasingly focused on the role of post-translational modifications, especially phosphorylation, in AS regulation [41–43]. For example, AMP-activated protein kinase (AMPK) and SR protein kinase 1 (SRPK1) phosphorylate SRSF1 [44, 45]; PPM1G phosphorylates SRSF3 [46]; CDK11 phosphorylates SF3B1 [47]; oncogenic KRAS regulates SFs phosphorylation [48]; SR protein kinase regulates the phosphorylation of the RSRSP stretch in RBM20 [49]; ERK2 regulates the phosphorylation of Thr113 and Thr118 [50]; and the C-terminal domain of RNA polymerase II (Pol II CTD) is also phosphorylated [51]. These phosphorylation events in SFs and RBPs have various regulatory effects on AS. Therefore, further exploration through phosphoproteomic analysis of CRC samples to map the phosphorylation states of SFs and RBPs could yield many interesting findings, providing a robust theoretical foundation for targeted splicing therapies in CRC.In recent years, miRNAs have emerged as a new research focus in CRC due to their strong association with the disease [52–56]. Dysregulated miRNAs can exert diverse effects on CRC, including promoting proliferation, invasion, apoptosis evasion, dysregulation of cell cycle, and angiogenesis promotion by regulating their target genes [56]. Moreover, miRNAs are also involved in crucial signaling pathways related to CRC such as Wnt/β-catenin, EGFR, TGFβ, and TP53 [52]. Our experimental data has revealed that many significantly different AS events are not only associated with the main genes at the splicing sites but also involve multiple miRNAs. This finding has sparked our interest. Based on the positional information of the splice sites of these AS events, we have observed that these miRNA binding sites are located within the boundaries of these AS events. Therefore, it can be inferred that during abnormal splicing, these AS events also impact the splicing of miRNA transcripts, thereby influencing the quantity and balance of miRNAs and potentially exerting varying degrees of impact on CRC. This suggests that in addition to directly targeting miRNAs for CRC treatment, an alternative approach could involve the identification of AS events that affect their expression and subsequently targeting these AS events to attenuate the production of oncogenic miRNAs, thus achieving therapeutic goals.

In addition to SF and miRNA, aberrations in oncogenes and tumor suppressor genes are also major factors contributors to the occurrence and progression of CRC. As a result, we place considerable emphasis on studying their abnormal AS events. Common oncogenes implicated in CRC include *KRAS*, *BRAF*, and *CDK4/6*, while *APC* and *TP53* are frequently observed tumor suppressor genes. Studies have shown that AS of *KRAS* transcripts results in the production of two isoforms, namely KRAS 4A and KRAS 4B, with selective inclusion of exon 4 [57]. Interestingly, both KRAS 4A and KRAS 4B exhibit oncogenic properties when *KRAS* is constitutively activated by mutations in exon 2 or 3 [58]. *CCND1* is closely associated with the cell cycle regulation in tumor cells, and one of its oncogenic isoforms, CCND1b, exerts distinct effects on CRC compared to its normal splicing isoform, CCND1a, due to the production of unique C-terminal protein sequences [59, 60]. AS isoforms of *APC*, such as cAPC and BS-APC, have demonstrated significant efficacy in inhibiting the growth of colon tumor cells. However, it has been observed that the isoform 0.3 APC lacks this function, indicating potential distinct roles of different *APC* subtypes in colorectal cancer development [8]. Similarly, different transcript variants with distinct tumorigenic and prognostic characteristics have been identified in *TP53* [19]. In summary, the significant impact on CRC is attributed to specific oncogenic/suppressor AS isoform. Therefore, effective targeted therapy would necessitate precise targeting of the isoforms unique to CRC. *CDK4* is a Cyclin-Dependent Kinase that functions by forming a complex with cyclin D, promoting cell cycle progression from G1 to S phase. Currently, *CDK4/6* inhibitors such as Palbociclib and Trilaciclib have been used in clinical settings for tumor treatment; however, their efficacy remains limited [17]. Notably, as associated partners of D-type cyclins, CDK4 and CDK6 exhibit multiple distinct functions in cancer [21]. Hence, we hypothesize that in CRC, aberrant AS events lead to the formation of diverse Cyclin D-CDK4/6 complexes, rendering CDK4/6 inhibitors imprecise in targeting the truly active complexes, thus failing to elicit an effective therapeutic response. Consequently, in our data analysis, we specifically focus on an exon 2 skipping event in CDK4.This leads to the production of a truncated CDK4 protein that coexists within the p27-CDK4-CCND1 complex. Furthermore, upon knocking down CDK4E2 in CRC using siRNA, we observed that more cells were arrested in the G1 phase, leading to a weakened proliferation capacity but enhanced migration capability. Therefore, our findings suggest that the suboptimal efficacy of *CDK4* inhibitors may be attributed to their lack of specificity towards CRC-specific isoforms. However, given the biased formation of the complex containing the truncated *CDK4* in CRC, it may be feasible to achieve targeted therapy exclusively against CRC by designing inhibitors that specifically target the p27-CDK4-CCND1 complex in CRC or by identifying upstream splicing factors that regulate the splicing of CDK4 exon 2. This approach would minimize adverse effects on normal tissues while selectively targeting the specific isoforms present in CRC.

## Conclusion

Within CRC, there exist a plethora of intricate and diverse AS events that lead to the formation of tumor-specific splicing products. Consequently, these distinct splicing products may exhibit both similarities and differences in their protein functionalities when compared to those observed in healthy cells. To advance targeted therapy for CRC, further investigation is imperative to explore the splicing products of target genes in CRC and comprehend their protein functionalities as well as pertinent information regarding upstream splicing factors. This will facilitate the development of protein inhibitors specifically targeting CRC-specific splicing variants or the regulation of upstream splicing factors, ultimately leading to precise targeted therapy for CRC.

### Supplementary Information

Additional file 1: Table S1. The 14,314 significantly different AS events (p<0.01) between CRC and normal tissues in the 16 samples.

## Data Availability

The data utilized in this research was obtained from the Gene Expression Omnibus database, series GSE138202 (https://www.ncbi.nlm.nih.gov/geo/query/acc.cgi?acc=GSE138202).
